# Emerging roles of polyunsaturated fatty acid synthesis pathway in colorectal cancer

**DOI:** 10.1080/19768354.2023.2189933

**Published:** 2023-03-20

**Authors:** Young-Ah Moon

**Affiliations:** Department of Molecular Medicine, Inha University College of Medicine, Incheon, South Korea

**Keywords:** colorectal cancer, polyunsaturated fatty acid, ELOVL5, FADS2, arachidonic acid

## Abstract

The development of colorectal cancer typically involves the accumulated influences of genetic alterations, medical issues, lifestyle, and diet. Dietary fatty acids appear to affect the tumorigenesis and progression of colorectal cancer. Despite conflicting results, the current consensus on the effects of very long-chain polyunsaturated fatty acids on colorectal cancer is that low levels of eicosapentaenoic acid and docosahexaenoic acid, and high levels of arachidonic acid are associated with an increased risk of colorectal cancer. Altered levels of arachidonic acid in membrane phospholipids can change the levels of prostaglandin E_2_, which affect the biological activities of cancer cells in multiple stages. Arachidonic acid and other very long-chain polyunsaturated fatty acids can affect tumorigenesis in prostaglandin E_2_-independent manners as well, including stabilization of β-catenine, ferroptosis, ROS generation, regulation of transcription factors, and de novo lipogenesis. Recent studies have revealed an association between the activities of enzymes synthesizing very long-chain polyunsaturated fatty acids and tumorigenesis and cancer progression, although the mechanisms are still unknown. In this study, PUFA effects on tumorigenesis, the endogenous very long-chain polyunsaturated fatty acid synthesis pathway, metabolites of arachidonic acid and their effects on tumorigenesis and progression of CRC, and current knowledge that supports the association of the enzymes involved in the polyunsaturated fatty acid synthesis pathway with colorectal cancer tumorigenesis and progression are reviewed.

## Introduction

Colorectal cancer (CRC) is the third most common cancer and second most common cause of cancer-related death globally (WHO 2022). Accumulation of various factors accounts for most cases of CRC. These factors include genetic alterations; medical issues, such as a family history of CRC, inflammatory bowel disease, obesity, and diabetes. Smoking, excessive alcohol consumption, physical activity, and dietary factors that include meat, vegetables, dietary fiber, and fish consumption are also critical factors in the development of CRC (Thanikachalam and Khan 2019; Li et al. 2021).

Dietary fats, especially dietary fatty acids, appear to affect he tumorigenesis and progression of CRC. Epidemiological studies have suggested that saturated fatty acids and ω-6 very long chain (VL)-polyunsaturated fatty acids (PUFAs) may enhance colorectal carcinogenesis, whereas ω-3 VL-PUFAs may have protective effects (Van Blarigan et al. 2018), although more studies are still needed to establish their effects and the mechanisms involved. Over the last decade, whole genome analysis and transcriptomic studies using CRC tissues and cell lines have revealed alterations in gene expression related to VL-PUFA synthesis and metabolism during tumorigenesis and progression of CRC, implicating these genes as therapeutic targets for cancer treatment, as well as markers to assess the severity of cancer or prognosis.

This review overviews PUFA effects on tumorigenesis, the endogenous VL-PUFA synthesis pathway and enzymes, metabolites of arachidonic acid and their effects on tumorigenesis and progression of CRC, expression changes of genes involved in VL-PUFA synthesis and metabolism, and epigenetic regulation of elongase of very long fatty acid 5 (ELOVL5) in cancer.

## Effects of PUFAs on tumorigenesis

Linoleic (C18:2, ω-6), α-linolenic acid (C18:3, ω-3), arachidonic acid (C20:4, ω-6), eicosapentaenoic acid (EPA, C20:5, ω-3), and docosahexaenoic acid (DHA, C22:6, ω-3) are the major components of membrane phospholipids. VL-PUFAs with more than 20 carbons (C20) also have various biological activities. They are ligands for nuclear receptors, including peroxisome proliferator-activated receptors and liver X receptors (LXRs) (Chambrier et al. 2002; Yoshikawa et al. 2002; Bordoni et al. 2006). VL-PUFAs are also regulators of transcription factors, such as sterol regulatory element binding protein-1 (SREBP-1) and carbohydrate element binding protein (ChREBP) (Hannah et al. 2001; Dentin et al. 2006). Their metabolites, which include eicosanoids and resolvins, act as signaling molecules in various physiological and pathological processes. Because of these essential roles, deficiencies in PUFAs result in disease conditions (Spector and Kim 2015). Conversely, many clinical and epidemiologic studies have revealed that VL-PUFAs, especially in the form of ω-3, which is rich in fish oil, have beneficial effects on the prevention and improvement of cardiovascular and metabolic diseases (Burns et al. 2018). The effects of VL-PUFAs on inflammation and cancer have not yet been clearly defined, and various studies have reported both pro-/anti-inflammatory and pro-/anti-tumorigenic roles (Hardman 2004; Hall et al. 2008; Calder 2017; Burns et al. 2018; Innes and Calder 2018; Bae et al. 2020; Dierge et al. 2021).

Population-based studies designed to determine the association between FA intake and CRC have demonstrated conflicting results, including a lack of association (Sasazuki et al. 2011; Sakai et al. 2012; Hodge et al. 2015). The influence of genetic variations in genes related to prostanoid synthesis, such as prostaglandin-endoperoxide synthase (PTGS) 1, PTGS2, arachidonate lipoxygenase (ALOX) 12, ALOX5, ALOX15, and 5-lipoxygenase activating protein, has been proposed as a reason for the inconsistent results of dietary VL-PUFAs on CRC risk (Habermann et al. 2013). Currently, the consensus on VL-PUFA on CRC seems to be that low levels of EPA and DHA, and high levels of arachidonic acid are associated with an increased risk of CRC (Pot et al. 2008). Similarly, dietary supplementation with ω-3 PUFA and ω-6 PUFA suppresses and enhances, respectively, the risk of CRC (Hardman 2004; Hall et al. 2008; Sasazuki et al. 2011; May-Wilson et al. 2017). Arachidonic acid seems to increase colon cancer cell growth, whereas DHA acts in another way (Habbel et al. 2009). Therefore, supplementation of ω-3 PUFA has been used in cancer treatment to minimize resistance and improve the efficacy of radiotherapy and chemotherapy (Hardman 2004; Dupertuis et al. 2007). One of the ways in which ω-6 arachidonic acid affects colon cancer cells may be mediated by the production of prostaglandins (PGs), whereas ω-3 PUFAs inhibit PG production from ω-6 PUFA (Dupertuis et al. 2007; Habbel et al. 2009). Independent of PG production, arachidonic acid could be pro-tumorigenic by stabilizing β-catenin through direct interaction with Fas-associated factor 1 (FAF1), thus stimulating tumor growth (Kim et al. 2015). The peroxidation of PUFAs induces oxidative stress in cells, which can be pro-inflammatory and pro-carcinogenic (Cai et al. 2012). In contrast, peroxidation of PUFAs can be cytotoxic and tumor-suppressive in CRC cells (Dupertuis et al. 2007). VL-PUFAs of both ω-6 and ω-3 can mediate anti-cancer effects through ferroptosis, an iron-dependent form of programed cell death (Dixon et al. 2012) characterized by the accumulation of lipid peroxides (Dierge et al. 2021). The conditions and mechanisms of the effects of ω-6 and ω-3 PUFAs on CRC are unclear, and further studies are required.

## Generation of endogenous VL-PUFAs

Linoleic and α-linolenic acids are essential fatty acids with 18 carbons. They cannot be synthesized in mammalian cells and must be supplied in the diet (Spector and Kim 2015). Longer chain PUFAs with more double bonds than linoleic and α-linolenic acid in the ω-6 and ω-3 series can be generated through reactions that are performed by FA elongation and desaturation systems on the endoplasmic reticulum (ER) membrane in cells (Cinti et al. 1992). Arachidonic acid, EPA, and DHA are representative VL-PUFAs generated from linoleic and α-linolenic acid through these systems (Spector and Kim 2015).

**Figure 1.**
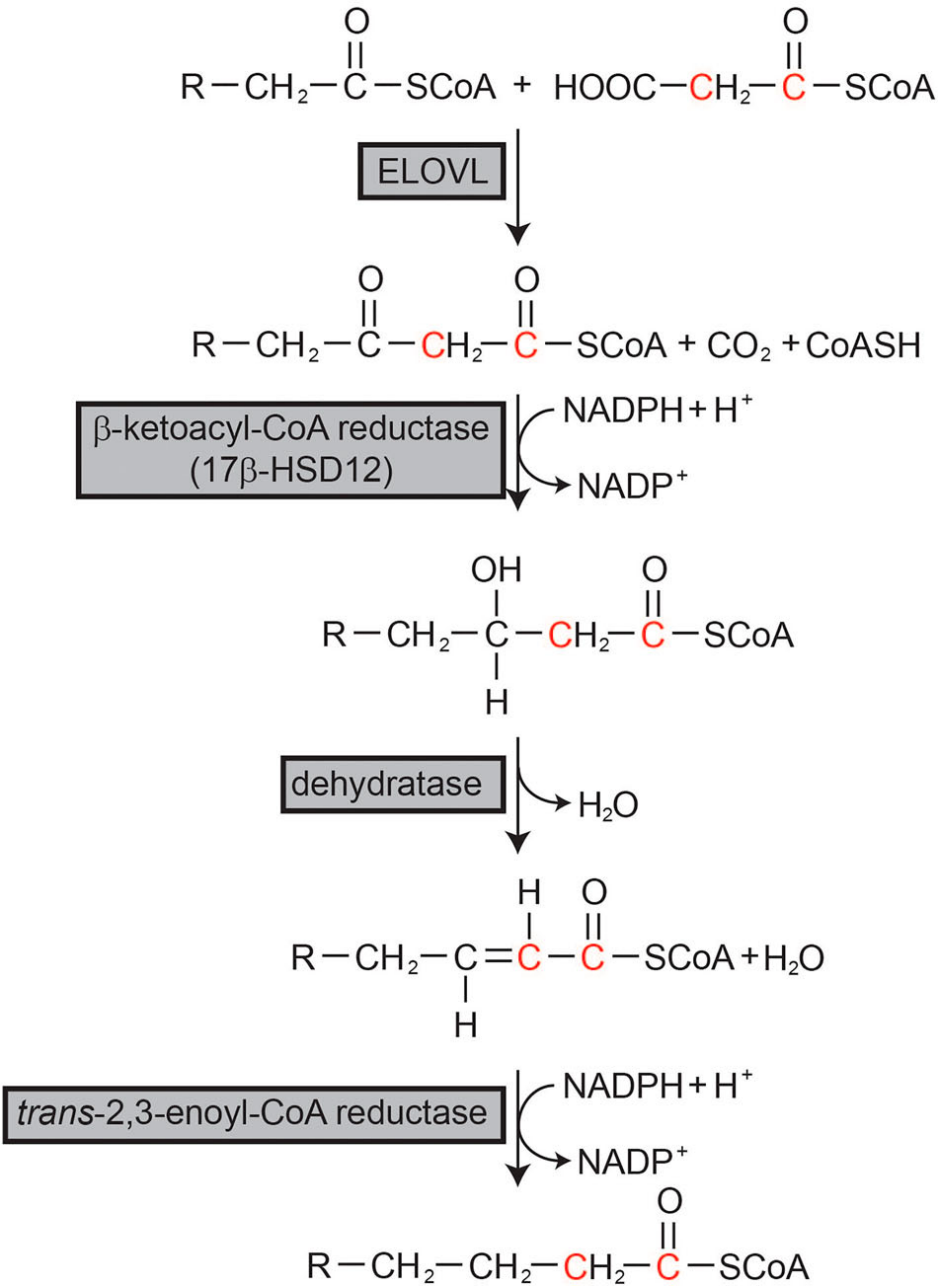
Fatty acid elongation system. Fatty acids longer than C18 are elongated through the fatty acid elongation system on the endoplasmic reticulum membrane. R represents the fatty acyl chain.

FAs longer than 18 carbons are generated by the FA elongation system on the ER membrane (Figure 1) (Cinti et al. 1992). This system adds two carbons to preexisting fatty acyl-CoA through a cycle of four consecutive reactions of condensation, reduction, dehydration, and reduction. Each reaction is catalyzed by a separate enzyme. Condensation between malonyl-CoA and preexisting fatty acyl-CoA to generate β-ketoacyl-CoA is catalyzed by ELOVLs. Next, β-ketoacyl reductase, also known as 17-beta hydroxysteroid dehydrogenase 12 (HSD17B12), catalyzes the generation of β-hydroxyacyl-CoA from β-ketoacyl-CoA using NADPH as a reducing cofactor. Next, *trans*-2,3-enoly-CoA is generated by the catalytic action of dehydratase, followed by another reduction catalyzed by *trans*-2,3-enoly-CoA reductase (TECR) using NADPH to generate the final 2-carbon elongated fatty acyl-CoA (Cinti et al. 1992). In this cycle, the condensation step catalyzed by ELOVLs is the rate-limiting step. Seven ELOVLs (ELOVL1, 2, 3, 4, 5, 6, and 7) exist in mammals (Jump 2009). Each ELOVL exhibits substrate specificity for fatty acyl-CoA with different chain lengths and numbers of double bonds, and is expressed in a tissue-specific manner. However, other enzymes that comprise the elongation system seem to possess no substrate specificity and are ubiquitously expressed in all tissues (Moon and Horton 2003).

**Figure 2.**
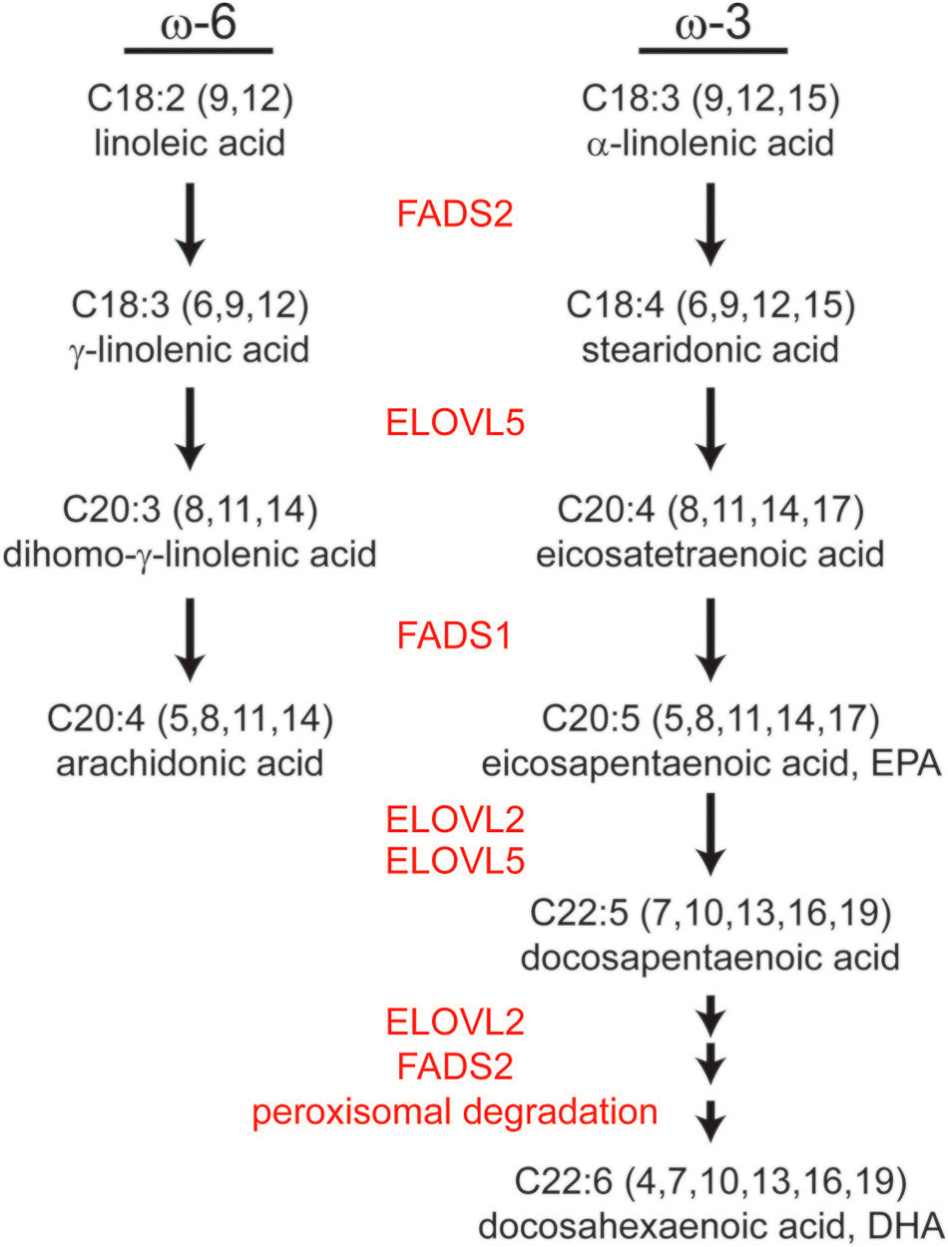
VL-PUFA synthesis pathway. Arachidonic acid, EPA, and DHA are the major VL-PUFAs synthesized from linoleic and α-linolenic acid by fatty acid elongases and desaturases that exist on the endoplasmic reticulum membrane.

To generate arachidonic acid, EPA, and DHA from linoleic and α-linolenic acid, ELOVL2, ELOVL5, fatty acid desaturase 1 (*Δ*5 desaturase; FADS1), and FADS2 (*Δ*6 desaturase) involve the reactions (Figure 2). Linoleic and α-linolenic acids are reduced to γ-linolenic acid and stearidonic acid by FADS2, followed by elongation to dihomo- γ-linolenic acid (C20:3, ω-6) and ETA (C20:4, ω-3) by ELOVL5. Following reduction by FADS1, arachidonic acid and EPA are generated. ELOVL2 is involved in the elongation of PUFAs longer than C20 and C22, and peroxisomal degradation may additionally occur to generate DHA (Jakobsson et al. 2006) (Figure 2). The importance of ELOVL5 in maintaining VL-PUFA in the cell was demonstrated in *Elovl5* knock out mice. The lack of ELOVL5 resulted in a reduction in cellular VL-PUFA content, and the phospholipid class displayed the greatest change (Moon et al. 2009).

## Metabolites of arachidonic acid and effects on cancer

Arachidonic acid is a precursor of C20 eicosanoids. Arachidonic acid at the *sn*-2 position of membrane phospholipids is released by phospholipase A2 and can be oxidized by the catalytic actions of cyclooxygenase (COX, also known as prostaglandin-endoperoxide synthase [PTGS]), P-450 epoxygenase, and lipoxygenase (LOX) (Patel et al. 2008). The COX pathway generates five primary prostanoids that include prostaglandin (PG) D_2_, PGE_2_, PGF_2α_, PGI_2_, and thromboxane A_2_, which are signaling molecules with short half-lives that act in a paracrine or autocrine manner (Patel et al. 2008). Prostanoids play important roles in general physiological and pathological processes, including inflammation, fever, smooth muscle constriction and relaxation, vascular constriction and relaxation, platelet aggregation, ovulation, and labor. The two major isoforms of COX (COX-1 and COX-2) act in the same fashion to generate prostanoids, but with different expression patterns in humans. COX-1 is constitutively expressed in most tissues, whereas COX-2 expression is induced in certain pathological conditions. Selective inhibition of COX-2 can reduce the side effects generated by inhibiting the physiological processes performed by COX-1 (Patel et al. 2008).

The eicosanoids produced are secreted into the extracellular microenvironment and bind to their specific receptors on the plasma membrane. PGD_2_ binds to prostaglandin D_2_ receptor (DP), PGE_2_ to prostaglandin E_2_ receptors (Eps), PGF_2α_ to prostaglandin F_2α_ receptor (FP), PGI_2_ to prostacyclin receptors (IPs), and TXA_2_ to thromboxane receptor (TBXA2R). Generally, PG pathways contribute to tumorigenesis by mediating cell proliferation, growth, apoptosis, invasion, migration, metastasis, and angiogenesis. Thus, PGs are critical mediators of cancer (Wang et al. 2015).

PGE_2_ is one of the major PGs produced by COX protein and the most abundant PG found in CRC (Cai et al. 2006; Wang et al. 2015). PGE_2_ binds to its specific receptors, EP1–EP4. These are G-protein-coupled receptors that transfer their signals via different signaling pathways. G proteins coupled with EPs give rise to second messengers that include cAMP, Ca^2+^, and inositol triphosphate (IP_3_) to initiate downstream signaling often associated with tumor growth and metastasis. The signaling includes the phosphoinositide 3-kinase (PI3K), mitogen-activated protein kinase (MAPK) pathway, NF-κB, and β-catenin/T-cell factor signaling (Wang et al. 2022). PGE_2_ effects on cancer cell proliferation and metastasis have been studied in cultured cells and in various types of human cancers, including CRC, breast cancer, lung cancer, liver cancer, gastrointestinal cancer, pancreatic cancer, and renal and urinary cancer (Jara-Gutiérrez and Baladrón 2021). PGE_2_ affects multiple stages of cancer, including carcinogenesis, tumor cell proliferation, invasion, and interaction between tumor and immune cells (Wang et al. 2015).

Chronic inflammation is an important risk factor for the development of CRC (Ogino et al. 2011; Cai et al. 2012). COX-2 expression is upregulated during inflammation and PGE_2_ synthesis is increased, which can alter cytokine balance and expression of PG receptors, as well as activate cell proliferation (Mutoh et al. 2006). PGE_2_ levels and expression of COX-2 are also increased in colon tumors compared with the surrounding normal tissue in mouse models and human patients (Sano et al. 1995). In the colon, PGE_2_ has been associated with polyp formation and tumorigenesis (Mutoh et al. 2006). In a rat colon tumor model induced by azoxymethane, the addition of PGE_2_ for 25 weeks significantly increased the incidence of tumors, suggesting that PGE_2_ is involved in tumorigenesis and that inhibition of the receptors or PGE_2_ synthesis can be targets for the prevention and treatment of CRC (Wang et al. 2005; Mutoh et al. 2006). Experimental evidence of the involvement of PGE_2_ in tumorigenesis and cancer metastasis has led to the use of COX inhibitors, including aspirin and nonsteroidal anti-inflammatory drugs (NSAIDs), to reduce PGE_2_ and produce anti-cancer effects (Dannenberg et al. 2001). Randomized controlled trials demonstrated that regular use of aspirin for >20 years could reduce the long-term incidence and mortality of CRC (Rothwell et al. 2010). Regular use of nonselective NSAIDs and selective COX-2 inhibitors over 10–15 years reduced the development of colorectal cancer by 40%–50%, and reduced the number and size of adenomas in patients with familial adenomatous polyposis (Wang et al. 2005). While NSAIDs reduced the risk of CRC associated with COX-2 overexpression, no effect was observed in CRC with weak or absent expression of COX-2 (Chan et al. 2007). Selective COX-2 inhibitors have been included in therapeutic strategies, either as a prophylactic or adjuvant treatment for chemotherapy or radiotherapy (Hashemi Goradel et al. 2019). These results suggest that PGE_2_ is important in CRC, and that the level of precursor arachidonic acid might affect PGE_2_ production.

## Enzymes involved in VL-PUPA synthesis in CRC

Reprograming of cellular metabolic processes is a feature of cancer cells that leads to changes in enzymes involved in de novo lipogenesis and FA profiles (Peck and Schulze 2016). Cellular changes occurring upon alteration in arachidonic acid content in membrane phospholipids have been demonstrated in endothelial cells. In the study, arachidonic acid content affected the viscosity of the cell membrane and thus cellular motion, and regulated cell adhesion and migration (Rossen et al. 2011). The increased arachidonic acid content in phosphatidylinositol at the cancer cell/stromal cell interface in CRC patients may imply the influence of arachidonic acid content in cancer progression (Hiraide et al. 2016). Moreover, arachidonic acid in membrane phospholipids is a source of PGE_2_, which affects multiple stages of cancer. As described earlier, VL-PUFAs other than arachidonic acid can also affect CRC. Therefore, changes in the activities of enzyme components in the VL-PUFA synthesis pathway could influence CRC cells in various ways, including PGE_2_-dependent and -independent manners. An inclusive list of published data of the association of ELOVL5, FADS2, and HSD17B12 with CRC and other types of cancers are shown in Tables 1 and 2, respectively.

**Table 1.** Association of genes in VL-PUFA synthesis with human colorectal cancer (CRC).

	Summary	Reference
*ELOVL5*		
	Genome-wide genotyping array of 3494 individuals with invasive CRC revealed SNPs at 6p12.1, where the nearest gene was ELOVL5. The strongest association was shown for rs209489 with poorer survival, especially in individuals with distant metastatic CRC.	(Phipps et al. 2015)
	Increased expression of ELOVL5 was found in tumor samples in a TCGA data set (GSE20931).	(Mokhtari et al. 2022)
	Transcription of ELOVL5 was downregulated through DNA hypermethylation in the low passage CRC cell lines with BRAF mutation isolated from cancer tissue.	(Boot et al. 2017)
	Colon cancer epithelial cells were isolated from colon cancer patients. Expression of fatty acid synthase, stearoyl-CoA desaturate, ELOVL2, and ELOVL5 were increased in the cancer cells. Significant changes in ratios of fatty acids in phospholipid classes were detected.	(Hofmanová et al. 2021)
*FADS2*		
	Increased expression of FADS2 was found in tumor samples in a TCGA data set (GSE20931).	(Mokhtari et al. 2022)
*HSD17B12*		
	Increased expression of HSD17B12 was found in tumor samples in a TCGA data set (GSE20931).	(Mokhtari et al. 2022)
	Increased risk of death and progression of colorectal cancer in patients with 10838164 C>T genetic variant was reported, which was correlated with increased transcriptional activity and upregulation of HSD17B12.	(Lin et al. 2020)

**Table 2.** Association of genes in VL-PUFA synthesis with human cancers other than CRC.

Cancer type	Summary	Reference
*ELOVL5*		
Gastric cancer	ELOVL5 expression was upregulated and led to ferroptosis sensitization in mesenchymal type gastric cancer cells	(Lee et al. 2020)
Breast cancer	Expression levels of ELOVL1, 5, and 6 were significantly upregulated in triple-negative tumors.	(Yamashita et al. 2017)
	Decreased expression of ELOVL5 and IGFBP6 was associated with poor prognosis.	(Shkurnikov et al. 2019)
	Knockdown of ELOVL5 and IGFBP6 genes increased the expression of matrix metalloproteinase 1 and decreased intercellular adhesion, suggesting more efficient invasion of tumor cells. Reduced expression of ELOVL5 and IGFBP6 genes in tumor cells could lead metastasis with a higher probability.	(Nikulin et al. 2021)
Prostate cancer	High expression of ELOVL5 was suggested as a potential marker of prostate cancer and higher incidence of metastasis.	(Romanuik et al. 2009)
	Potent and direct androgen receptor-mediated induction of ELOVL5 was presented in prostate cancer cell. Patient-derived cells revealed that ELOVL5 expression was upregulated in prostate cancer compared with nonmalignant prostate.	(Centenera et al. 2021)
Renal cell carcinoma	High level of ELOVL5 correlated with higher clinical staging and poor clinical prognosis. High expression of ELOVL5 was negatively associated with overall survival in a TCGA database.	(Nitta et al. 2022)
	Knockout of ELOVL5 in the cancer cells suppressed proliferation and induced apoptosis in ACHN and 786-O cancer cells. The knockout inhibited in vivo tumor growth.	(Nitta et al. 2022)
*FADS2*		
Breast cancer	FADS2 activity was increased in cancerous tissue. Cancerous tissue contained higher levels of C20:3, ω-6 and arachidonic acid than adjacent noncancerous tissue did. It was associated with PGE_2_ level especially in estrogen receptor-negative cancer.	(Pender-Cudlip et al. 2013)
*HSD17B12*		
Breast cancer	Immunoreactivity of HSD17B12 was significantly associated with poor prognosis of patients.	(Nagasaki et al. 2009)
	RNA interference of HSD17B12 resulted in COX-2 dependent growth inhibition in SK-BR-3 cancer cells. Cell growth was recovered by addition of arachidonic acid.	(Nagasaki et al. 2009)
	Knockdown of HSD17B12 increased proliferation and migration of MCF7 and MDA-MB-453 cells.	(Tsachaki et al. 2020)
	Knockdown of HSD17B12 decreased proliferation of SUM159 cells.	(Tsachaki et al. 2020)
Ovarian cancer	HSD17B12 expression in cancer tissue was suggested as a marker of poor prognosis. Silencing of HSD17B12 in cancer cell lines resulted in growth inhibition and increased apoptosis.	(Szajnik et al. 2012)
	Increased expression was detected in cancer tissue by immunostaining and was associated with cancer severity.	(Kemiläinen et al. 2018)

**Figure 3.**
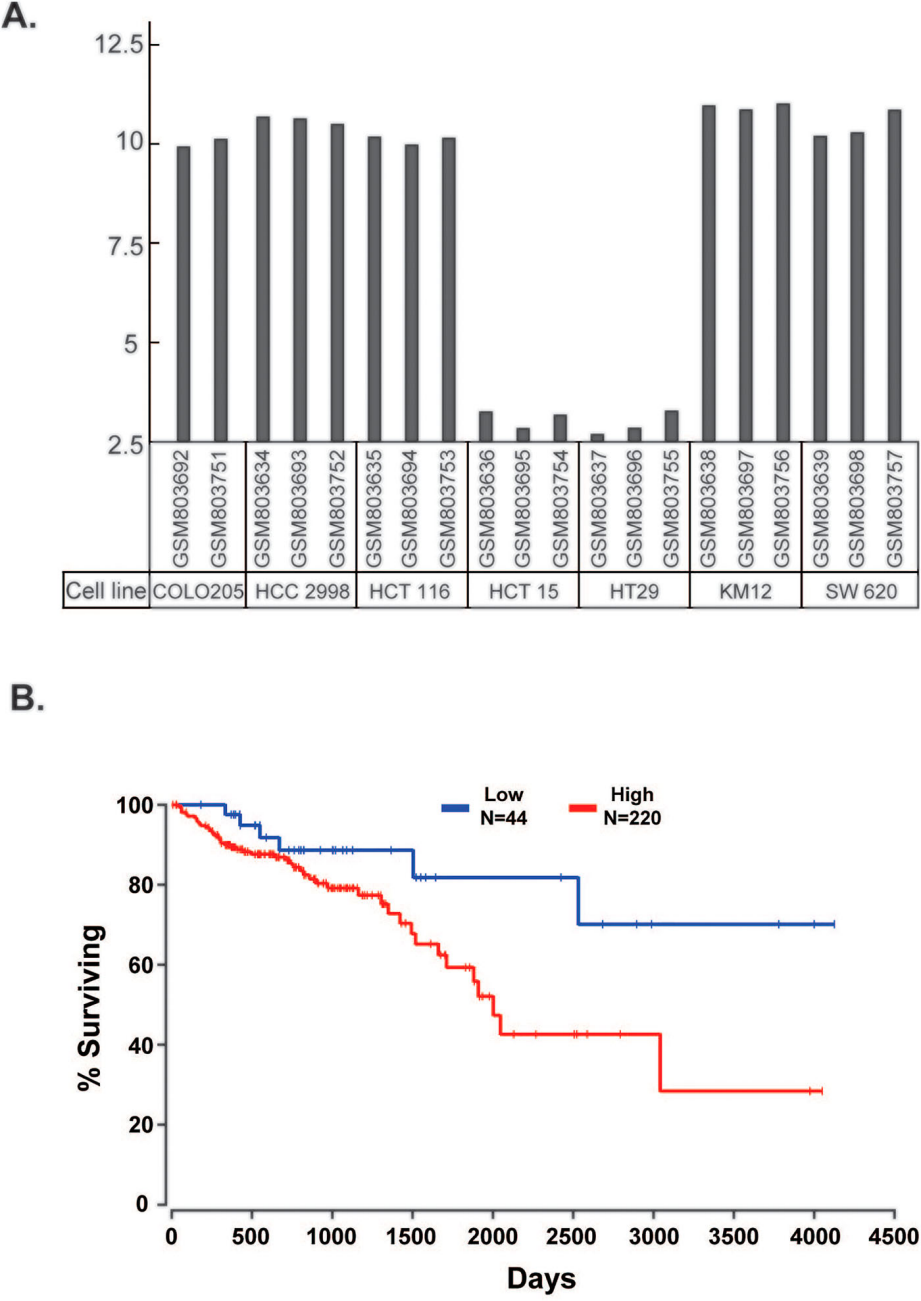
Association of ELOVL5 with colorectal cancer (CRC). (A) ELOVL5 expression in the indicated CRC cell lines (GEO profile GDS4296). (B) TCGA survival data that link ELOVL5 expression level with overall survival of the patients of colon adenocarcinoma shows that the patients with low expression of ELOVL5 (<10th percentile) showed a better survival rate than those with high expression of ELOVL5 (>50th percentile).

*ELOVL5*: ELOVL5 activity is essential for maintaining VL-PUFA levels in phospholipids, as shown in a study involving *Elovl5^−/−^* mice (Moon et al. 2009). Therefore, changes in the expression of ELOVL5 in cancer cells would change the VL-PUFA profile and affect the proliferation and progression of cancer cells. According to a Gene Expression Omnibus (GEO) profile (GDS4296) using various CRC cell lines, COLO205, HCC 2998, HCT116, KM12, and SW 620 cells exhibited high expression of ELOVL5, whereas ELOVL5 expression was negligible in HCT 15 and HT 29 cells (Figure 3(A)). ELOVL5 expression in CRC is inversely associated with the prognosis of patients with CRC. The Cancer Genome Atlas (TCGA) survival data that linked ELOVL5 expression level with overall survival of patients with colon adenocarcinoma revealed better survival rates for patients with low expression of ELOVL5 (<10th percentile) compared to patients with high expression of ELOVL5 (>50th percentile) (Figure 3(B)). Genome analysis has demonstrated increased expression of ELOVL5 in human CRC and other types of cancers, including breast, prostate, and kidney cancers. The findings suggest the involvement of ELOVL5 in cancer cell proliferation and invasion, although its effects and mechanisms remain unclear. Study data on the association between ELOVL5 expression and cancers are presented in Tables 1 and 2.

Gene expression analysis of CRC using GEO dataset GSE20931 showed that the expression of ELOVL5, FADS2, HSD17B12, and *trans*-2,3-enoyl-CoA reductase (TECR), the enzymes involved in PUFA elongation and desaturation, were significantly increased in colon cancer samples compared to the normal tissue. The findings suggest that changes in PUFA contents could affect cancer cell behavior (Mokhtari et al. 2022). In another study, changes in the expression of genes involved in de novo FA and VL-PUFA synthesis were detected in cultured colon cancer cells. Increased expression of fatty acid synthase, stearoyl-CoA desaturase, FADS2, and ELOVL5 in tumor epithelial cells was correlated with changes in fatty acid contents of cellular phospholipids, which reflected the increased activities of ELOVL5/2 and FADS2 in these tumor cells (Hofmanová et al. 2021). One of the mechanisms that regulates ELOVL5 activity may be related to genetic variation. A genome-wide analysis found an association between the single nucleotide polymorphisms (SNP) near 6p12.1/ELOVL5 gene and survival outcomes in patients with distant metastatic CRC. The SNP, rs2309489, exhibited the strongest association with poor survival rate. However, the role of genetic variation in CRC has not been elucidated, and the use of the SNP as a prognostic marker has not yet been determined (Phipps et al. 2015). Upregulation of enzymes involved in PUFA elongation has also been reported in other types of cancer. In a study that compared gene expression in 74 breast cancer tissues with that in normal breast tissues, ELOVL1, 5, and 6 were detected as genes that were upregulated in tumors (Yamashita et al. 2017).

Mechanisms of how ELOVL5 activity affects cancer cells have been suggested in a few studies performed in prostate cancer, renal cell cancer, and gastric cancer cells. In prostate cancer cells, ELOVL5 expression was induced by androgen, and increased expression of ELOVL5 in prostate cancer was demonstrated in cultured cells, xenografts, and clinical tumors of prostate cancer (Centenera et al. 2021). Upon depletion of ELOVL5 in prostate cancer cells, the cells exhibited morphological and functional changes in the mitochondria, resulting in the excess generation of ROS to kill the cells (Centenera et al. 2021). In renal cell cancer, higher levels of ELOVL5 correlate with poor clinical prognosis, and ELOVL5 seems to lead to cancer cell proliferation and invasion (Nitta et al. 2022). FA changes due to the increased activity of ELOVL5 have been related to increased cell proliferation and invasion (Nitta et al. 2022). Protein kinase B-mammalian target of rapamycin-signal transducer and activator of transcription 3 (AKT-mTOR-STAT3) signaling through AKT Ser473 phosphorylation has been suggested as a mechanism. However, more studies are needed to determine how these lipid changes affect signaling pathways.

Recently, ferroptosis has emerged as a mechanism of programed cell death. Ferroptosis is characterized by the accumulation of peroxided VL-PUFAs. Some gastric cancer cells exhibit differential expression of ELOVL5 and FADS1 through changes in DNA methylation of their promoter regions. ELOVL5 expression is associated with the levels of cellular arachidonic acid and adrenic acid (C22:4, ω-6) in cancer cells, which are subjected to lipid peroxidation and ferroptosis (Lee et al. 2020). As the ferroptosis pathway is a possible target for cancer treatment (Dierge et al. 2021), ELOVL5 could be a marker for determining whether ferroptosis-mediated treatment is applicable (Lee et al. 2020). The association between ferroptosis sensitization and differential expression of ELOVL5 needs to be determined to indicate its’ therapeutic feasibility in CRC.

Contrary to the results presented above, a study related the low expression of ELOVL5 and insulin-like growth factor binding protein 6 (IGFBP6) with pronounced metastasis in breast cancer (Nikulin et al. 2021). Inhibition of the ELOVL5 and IGFBP6 genes in breast cancer cells results in the increased expression of matrix metalloproteinase 1 and reduction of intercellular contacts. These changes in turn result in a more efficient invasion of tumor cells and higher probability of increased metastasis (Nikulin et al. 2021).

The studies presented above suggest that the cellular VL-PUFA composition and the activities of the synthesis pathway could play important roles in the development and progression of cancer. Although the functions of ELOVL5 and its mechanisms in tumorigenesis are still unknown, ELOVL5 could be a possible diagnostic and prognostic marker and a targetable molecule for CRC treatment.

*FADS2*: FADS2, a *Δ*-6 desaturase, performs the first rate-limiting step in the endogenous pathway to synthesize arachidonic acid from C18 PUFA (Figure 2) (Tang et al. 2003). The possible involvement of FADS2 in the pathogenesis of breast cancer has been suggested. Along with increased FDAS2 activity, levels of metabolites from linoleic acid, such as C18:3, ω-6, C20:3, ω-6, and arachidonic acid, as well as PGE_2_ levels, are reportedly increased in cancerous tissue compared to adjacent noncancerous tissue in breast tumors (Pender-Cudlip et al. 2013). These results may lead to similar effects of increased ELOVL5 expression, which leads to increased arachidonic acid production due to the greater conversion from linoleic acid. The correlation between FADS2 expression and PGE_2_ levels suggests that the enzyme activity toward arachidonic acid synthesis could affect PGE_2_ levels and, thus, tumorigenesis (Pender-Cudlip et al. 2013).

*HSD17B12*: HSD17B12, or β-ketoacyl-CoA reductase, is the enzyme involved in the second step of the FA elongation cycle (Figure 1) (Moon and Horton 2003). Therefore, overall FA elongation and cellular FA profiles can be affected by HSD17B12 activity. However, the enzyme’s mechanism in tumorigenesis is unknown; several studies have reported an association between HSD17B12 expression and cancer outcomes.

Higher expression of HSD17B12 was detected in colorectal tumor tissues, suggesting a possible correlation between its activity and CRC. Functional genetic variants of HSD17B12 are correlated with the outcome of CRC (Lin et al. 2020). A Cox regression model that evaluated the genetic effects on CRC overall survival and progression-free survival revealed that rs10838164 C>T in HSD17B12 was significantly associated with an increased risk of CRC progression and death. The T allele can increase HSD17B12 expression by enhancing the binding affinity of transcription factors to promote the transcriptional activity of the HSD17B12 gene (Lin et al. 2020).

Changes in the expression of HSD17B12 have been reported in ovarian and breast cancers (Kemiläinen et al. 2018; Tsachaki et al. 2020). High expression of HSD17B12, along with increased COX-2 expression, is associated with high-grade epithelial ovarian cancer (Kemiläinen et al. 2018). The expression level of HSD17B12 correlated with the severity of ovarian cancer, and its expression mimicked COX-2 expression, indicating its role in increased arachidonic acid and PGE_2_ production during ovarian cancer progression (Kemiläinen et al. 2018). High immunoreactivity in breast cancer has been significantly associated with poor prognosis of patients (Nagasaki et al. 2009). When HSD17B12 was knocked down in cancer cells with high expression of HSD17B12, cell growth was significantly inhibited with reduced total amounts of FAs and arachidonic acid. These changes were completely reversed by the addition of arachidonic acid. HSD17B12 activity may be correlated with PG production (Nagasaki et al. 2009). The biological significance and function of 17BHSD12 in human cancer remain unknown, and further studies are needed to elucidate its mechanism.

## Epigenetic regulation of ELOVL5 in CRC

Epigenetic regulation is one of the mechanisms that regulate downstream gene expression. Approximately 70% of genes contain CpG islands in their promoters (Saxonov et al. 2006; Deaton and Bird 2011). While unmethylated regions usually serve as transcriptional initiation sites, their methylation can form heterochromatin that inhibits the interaction with transcription factors or chromatin remodelers, leading to the inhibition of downstream gene expression (Saxonov et al. 2006; Deaton and Bird 2011). Many tumors exhibit changes in the methylation status of CpG islands during tumorigenesis (Paweł and Maria Małgorzata 2022). In a subset of colorectal tumors, an exceptionally high frequency of methylation of some CpG islands has been described and categorized as a ‘CpG island methylator phenotype (CIMP)’, where BRAF mutations are present in most cases (Ogino et al. 2011; Boot et al. 2017). CIMP is one of the mechanisms that lead to chromatin instability and microsatellite instability during tumorigenesis of CRC (Ogino et al. 2011).

Methylation-associated transcriptional repression has emerged as a mechanism that inhibits the expression of ELOVL5 in CRC cell lines (Boot et al. 2017). When DNA methylation and gene expression profiles were generated in CRC cell lines and cell lines with BRAF mutations, one of the subsets was reportedly associated with CIMP. ELOVL5 was hypermethylated along with family with sequence similarity 127 member B (FAM127B), mitochondrial transcription termination factor 1 (MTERF1), and zinc finger protein 606 (ZNF606) genes whose expressions were low (Boot et al. 2017). These findings suggest that ELOVL5 expression can be epigenetically regulated in CRC cells. A survival analysis using TCGA data showed that ELOVL5 hypermethylation was associated with improved overall survival, suggesting that low expression of ELOVL5 by DNA hypermethylation is protective in the progression of CRC progression (Boot et al. 2017). The authors also described the correlation of ELOVL5 expression with tumor stage and relapse-free survival. However, a mechanism that can explain this correlation has yet to be elucidated; changes in lipogenesis, the downstream transcriptional effect of the MAPK pathway, and its effects on apoptosis have been proposed.

## Conclusion

The roles of PGE_2_ and the responsible enzyme (COX-2) in the stages of CRC have been actively studied. The enzymes in the VL-PUFA synthesis pathway, including ELOVL5, FADS2, and HSD17B12, can affect cellular arachidonic acid level and PGE_2_ level, and thus could change the biological activities of cancer cells in multiple stages. Arachidonic acid and other VL-PUFAs can affect tumorigenesis in PGE_2_-independent manners as well, including stabilization of β-catenine, ferroptosis, ROS generation, regulation of transcription factors, and de novo lipogenesis. Recent studies have revealed an association between the activities of enzymes synthesizing VL-PUFA and tumorigenesis and cancer progression, although the mechanisms are still unknown. More studies are needed to elucidate the role of these enzymes and further their use as diagnostic and prognostic markers, and as therapeutic targets for CRC.
